# Percutaneous Nephrostomy versus Ureteral Stent for Severe Urinary Tract Infection with Obstructive Urolithiasis: A Systematic Review and Meta-Analysis

**DOI:** 10.3390/medicina60060861

**Published:** 2024-05-24

**Authors:** Young Joon Moon, Dae Young Jun, Jae Yong Jeong, Seok Cho, Joo Yong Lee, Hae Do Jung

**Affiliations:** 1 Department of Medicine, Graduate School, Yonsei University, Seoul 03722, Republic of Korea; jjunny72@gmail.com; 2 Department of Urology, Severance Hospital, Urological Science Institute, College of Medicine, Yonsei University, Seoul 03722, Republic of Korea; dyjun881101@yuhs.ac (D.Y.J.); joouro@yuhs.ac (J.Y.L.); 3 Department of Urology, National Health Insurance Service Ilsan Hospital, Goyang 10444, Republic of Korea; urojjy@nhimc.or.kr; 4 Department of Urology, Inje University Ilsan Paik Hospital, College of Medicine, Inje University, Goyang 10380, Republic of Korea; seokcho@paik.ac.kr; 5 Center of Evidence Based Medicine, Institute of Convergence Science, Yonsei University, Seoul 03722, Republic of Korea

**Keywords:** percutaneous nephrostomy, stent, ureteral obstruction

## Abstract

*Background and Objectives*: The European Association of Urology guidelines on urolithiasis highlight the limited evidence supporting the superiority of percutaneous nephrostomy (PCN) over retrograde ureteral stent placement for the primary treatment of infected hydronephrosis secondary to urolithiasis. We, therefore, conducted a systematic review and meta-analysis comparing the effects of PCN and retrograde ureteral stent in patients with severe urinary tract infections secondary to obstructive urolithiasis. *Materials and Methods*: Meta-analyses were performed to compare four outcomes: time for the temperature to return to normal; time for the white blood cell (WBC) count to return to normal; hospital length of stay; and procedure success rate. After a full-text review, eight studies were identified as relevant and included in our systematic review and meta-analysis. *Results*: No significant difference was detected between PCN and retrograde ureteral stenting for the time for the temperature to return to normal (*p* = 0.13; mean difference [MD] = −0.74; 95% confidence interval [CI] = −1.69, 0.21; I^2^ = 96%) or the time for the WBC count to return to normal (*p* = 0.24; MD = 0.46; 95% CI = −0.30, 1.21; I^2^ = 85%). There was also no significant difference between methods for hospital length of stay (*p* = 0.78; MD = 0.45; 95% CI = −2.78, 3.68; I^2^ = 96%) or procedure success rate (*p* = 0.76; odds ratio = 0.86; 95% CI = 0.34, 2.20; I^2^ = 47%). *Conclusions*: The clinical outcomes related to efficacy did not differ between PCN and retrograde ureteral stenting for severe urinary tract infection with obstructive urolithiasis. Thus, the choice between procedures depends mainly on the urologist’s or patient’s preferences.

## 1. Introduction

The prevalence of urolithiasis is increasing significantly on a global level, posing a substantial challenge to public health [1]. In the United States, the incidence of urolithiasis increased by approximately two-fold in the past 15 years. Approximately 1 in 11 individuals have experienced at least one episode of urolithiasis [2]. The estimated lifetime prevalence of urolithiasis in Europe and the United States ranges from 5% to 12%. Urolithiasis affects approximately 13% of male and 7% of female patients [3].

Urosepsis is one of the most severe complications associated with urolithiasis [4]. Obstructive pyelonephritis secondary to urolithiasis is considered a critical condition in the field of urology, necessitating prompt intervention to prevent urosepsis and mortality [5]. Immediate decompression of the pelvicalyceal system and expeditious initiation of antibiotic therapy are imperative initial strategies to prevent death in patients with urinary tract obstruction, as urosepsis is an independent risk factor for septic shock and mortality. Emergency decompression can be achieved through two methods: retrograde ureteral stent placement or percutaneous nephrostomy (PCN) [6,7].

Recent guidelines established by the European Association of Urology (EAU) state that prompt decompression is frequently required to mitigate the potential complications associated with infected hydronephrosis resulting from unilateral or bilateral renal obstruction caused by stones [8,9]. Retrograde placement of an indwelling ureteral stent and percutaneous placement of a nephrostomy tube are both recommended procedures for urgent decompression. Limited evidence supports the superiority of PCN over retrograde ureteral stent as the primary treatment for infected hydronephrosis [10].

Consequently, this systematic review with a meta-analysis was conducted to compare the effectiveness of PCN versus retrograde ureteral stent placement for the treatment of severe urinary tract infections (UTIs) associated with obstructive urolithiasis.

## 2. Materials and Methods

This systematic review with a meta-analysis is registered in PROSPERO under the registration number CRD42022336928. It was conducted and reported in accordance with the Preferred Reporting Items for Systematic Reviews and Meta-Analyses guidelines (Supplement Table S1).

### 2.1. Inclusion Criteria

Studies were assessed for inclusion in this systematic review with a meta-analysis by taking into account the participants, interventions, comparators, outcomes, and study design (i.e., PICOS) of the study. The patient population comprised individuals with severe UTI secondary to obstructive urolithiasis. The intervention was PCN, and the comparator was retrograde ureteral stent placement. The outcomes were the time for the temperature to return to normal, the time for the white blood cell (WBC) count to return to normal, the hospital length of stay, and the procedure success rate.

### 2.2. Search Strategy

A comprehensive review of scholarly articles published prior to March 31 2023 was conducted utilizing various academic databases, including PubMed, EMBASE, Cochrane Library, Web of Science, and Google Scholar. Manual cross-referencing searches were also conducted to identify potentially eligible studies that were not retrieved through the computer-based searches. Presentations at relevant medical meetings were also reviewed. The search strategies employed a variety of MeSH terms and keywords, including percutaneous nephrostomy, PCN, ureteral stent, stenting, obstructive uropathy, urolithiasis, and ureter stone (Supplement Table S2).

### 2.3. Data Extraction

The titles and abstracts of all the articles identified through the search strategies were screened for potential inclusion by two researchers (YJM and DYJ). The full text of each potential article was then independently evaluated by these two researchers to identify articles that may be relevant. The researchers extracted the most pertinent articles for each study and recorded the following details: first author, publication year, country, patient number and characteristics, inclusion and exclusion criteria, study design, procedures, and results. Disagreements were resolved through discussion until consensus was achieved or through arbitration facilitated by an impartial researcher (HDJ).

### 2.4. Quality Assessment for Studies

The Cochrane risk of bias (ROB) tool was employed to assess the risk of bias in randomized controlled trials (RCTs), while the methodological index for nonrandomized studies (MINORS) was utilized to evaluate the quality of nonrandomized studies. The level of evidence of each study was rated according to the Scottish Intercollegiate Guidelines Network (SIGN) checklist, which encompasses a range of research methodologies, including systematic reviews, meta-analyses, RCTs, cohort studies, case–control studies, diagnostic studies, and economics studies. Two researchers (YJM and DYJ) conducted independent quality assessments. After engaging in discussion with a third researcher (HDJ), all discrepancies regarding quality assessment were resolved.

### 2.5. Heterogeneity Tests

The presence of heterogeneity was evaluated using the Q statistic and Higgins’ I^2^ statistic [11]. The Q statistic was employed to assess statistical heterogeneity, while I^2^ was utilized to quantify the extent of heterogeneity. The I^2^ statistic developed by Higgins quantifies the proportion of overall variability attributed to heterogeneity (rather than random variation) among studies. Higgins’ I^2^ is calculated as follows, with Q representing Cochran’s heterogeneity statistic and df denoting the degrees of freedom:

$$I^{2}=\frac{Q-df}{Q}\times 100\%$$

Q statistic values with a *p* value < 0.10 indicated the presence of significant heterogeneity [12]. I^2^ values ≥ 50% were considered indicative of significant heterogeneity. When the I^2^ value was <50%, fixed-effect models were employed for a meta-analysis; when it was ≥50%, random-effect models were utilized. The assessment of studies yielding positive results involved the determination of pooled specificity, accompanied by 95% confidence intervals (CIs).

### 2.6. Statistical Analysis

Odds ratios (ORs) were utilized to quantify the treatment effects for dichotomous outcomes, while mean differences (MDs) were employed for the continuous outcomes. Both measures were accompanied by 95% CIs. Sensitivity analyses were conducted to ascertain whether the observed heterogeneities were attributed to a low study quality. The meta-analysis findings are displayed using forest plots. Publication bias was assessed with funnel plots and Egger’s test. All the statistical analyses were conducted using the R software (version 4.1.2, R Foundation for Statistical Computing, Vienna, Austria; http://www.r-project.org).

## 3. Results

### 3.1. Eligible Studies

**Figure 1:**
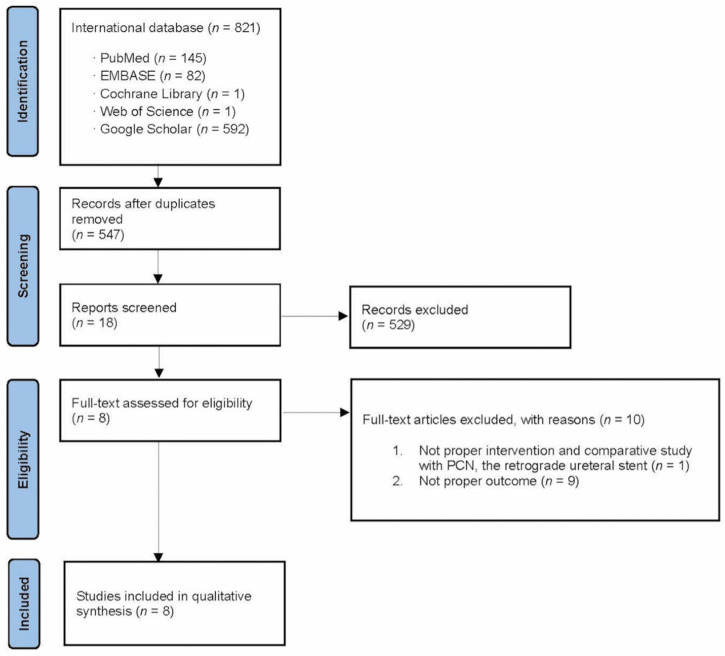
Study flow chart.

Our comprehensive examination of the available literature identified 821 studies for potential inclusion in this systematic review with a meta-analysis. Eight of these studies were ultimately deemed pertinent to the present investigation and chosen for inclusion in this systematic review with a meta-analysis (Figure 1) [13,14,15,16,17,18,19,20].

### 3.2. Characteristics of the Included Studies

Table 1 displays the attributes of the eight studies included in this systematic review with a meta-analysis [13,14,15,16,17,18,19,20]. All the studies compared the outcomes of patients who underwent PCN versus ureteral stent placement for urgent decompression in patients with severe UTI resulting from obstructive urolithiasis. The studies were published between October 1998 and August 2022. Four studies were conducted in various Asian countries, including China, Japan, Singapore, and Taiwan [13,14,16,17,18]; two were performed in the United States [17,19]; one was performed in Canada [15]; and one was conducted in Turkey [20]. The Canadian study was available only in abstract form [15].

### 3.3. Quality Assessment

**Figure 2:**
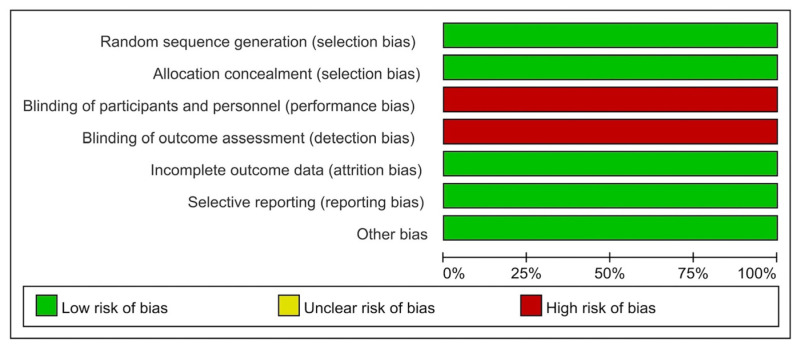
Risk of bias graph.

**Figure 3:**
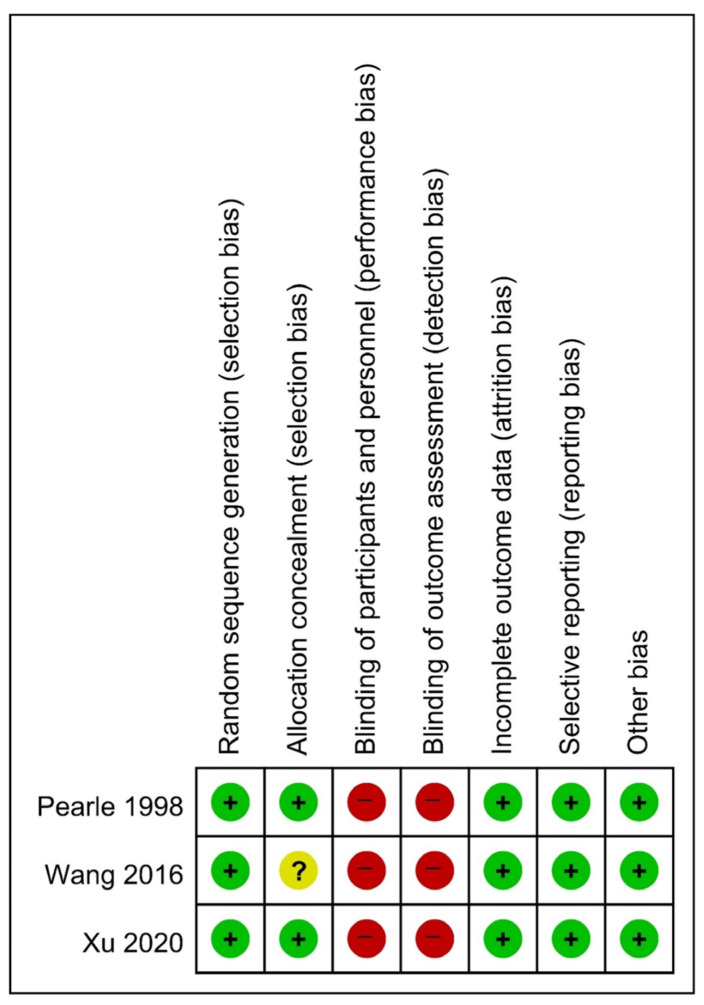
Risk of bias summary [14,16,17].

The results of the quality assessment of the included studies are presented in Table 1 and were determined to be satisfactory. Based on the SIGN checklist, the studies were rated as 1+ (n = 3), 2+ (n = 3), or 2− (n = 2). The ROB assessments for RCTs are presented in Figure 2 and Figure 3 [14,16,17]. Table 2 shows the MINORS scores for the non-RCTs [13,15,18,19,20]. All the included studies exhibited a moderate level of potential bias.

### 3.4. Publication Bias and Heterogeneity Assessments

**Figure 4:**
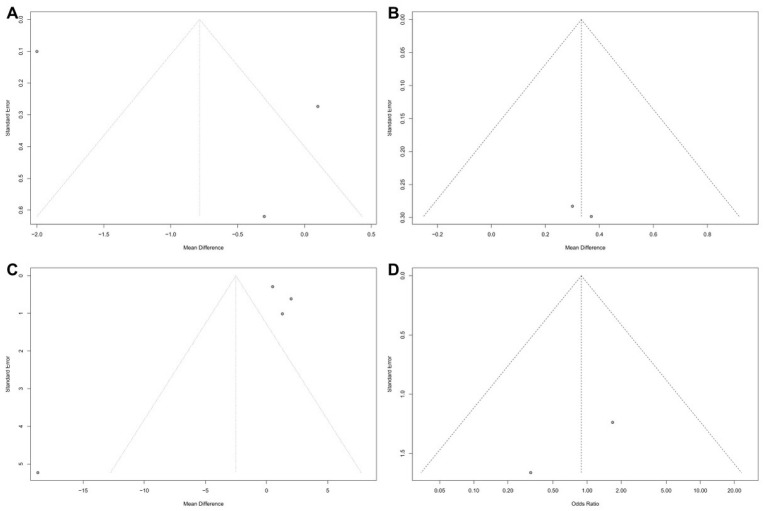
Funnel plots: (**A**) time for the temperature to return to normal, (**B**) time for the WBC count to return to normal, (**C**) hospital length of stay, and (**D**) procedure success rate.

Figure 4 displays the funnel plots for each outcome. The plots reveal minimal publication bias for the time for the WBC count to return to normal (Figure 4B) and the procedure success rate (Figure 4D). Publication bias was observed for other outcomes (time for the temperature to return to normal [Figure 4A] and hospital length of stay [Figure 4C]). Nevertheless, Egger’s test for the latter two outcomes showed no evidence of publication bias (*p* = 0.71 and *p* = 0.61, respectively).

Heterogeneity was minimal for the time for the WBC count to return to normal (*p* = 0.86, I^2^ = 0%) and the procedure success rate (*p* = 0.42, I^2^ = 0%). Thus, fixed-effect models were used to compare these outcomes between PCN and retrograde ureteral stent placement. By contrast, significant heterogeneity was found for the time for the temperature to return to normal (*p* < 0.01, I^2^ = 97%) and the hospital length of stay (*p* = 0.0003, I^2^ = 84%). Thus, random-effect models were used to compare these outcomes between PCN and retrograde ureteral stent placement.

**Figure 5:**
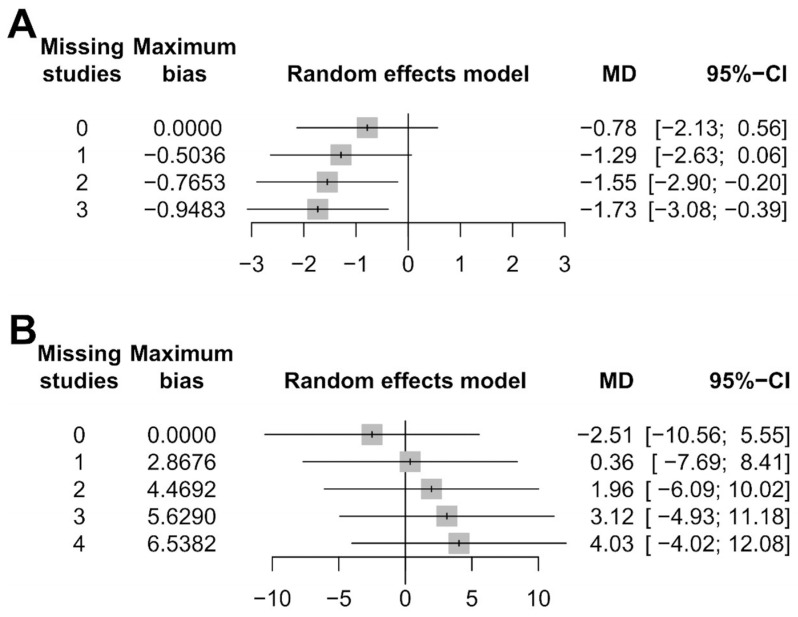
Sensitivity analysis for outcome-reporting bias: (**A**) time for the temperature to return to normal, and (**B**) hospital length of stay.

We also conducted sensitivity analyses for outcome-reporting bias to examine the effects of heterogeneity. The sensitivity of the meta-analysis for the time for the temperature to return to normal was considered robust, as the direction of the results was not affected when up to three studies were excluded (Figure 5A). The sensitivity of the meta-analysis for hospital length of stay results was less robust, as the direction of the results was affected when one study was excluded (Figure 5B).

### 3.5. Time for the Temperature to Return to Normal

**Figure 6:**
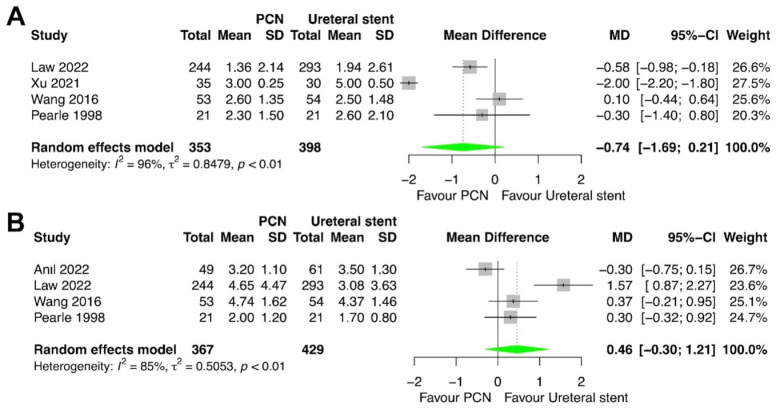
Forest plots: (**A**) time for the temperature to return to normal, and (**B**) time for the white blood cell count to return to normal [14,16,17,18,20].

The time for the temperature to return to normal was compared between PCN and retrograde ureteral stent placement in four studies [14,16,17,18]. The meta-analysis revealed no statistically significant difference between patients treated with PCN and those treated with retrograde ureteral stent placement for this outcome (*p* = 0.13; MD = −0.74; 95% CI = −1.69, 0.21; I^2^ = 96%) (Figure 6A).

### 3.6. Time for the WBC Count to Return to Normal

The time for the WBC count to return to normal was compared between PCN and retrograde ureteral stent placement in four studies [16,17,18,20]. The meta-analysis revealed no statistically significant difference between patients treated with PCN and those treated with retrograde ureteral stent placement for this outcome (*p* = 0.24; MD = 0.46; 95% CI = –0.30, 1.21; I^2^ = 85%) (Figure 6B).

### 3.7. Hospital Length of Stay

**Figure 7:**
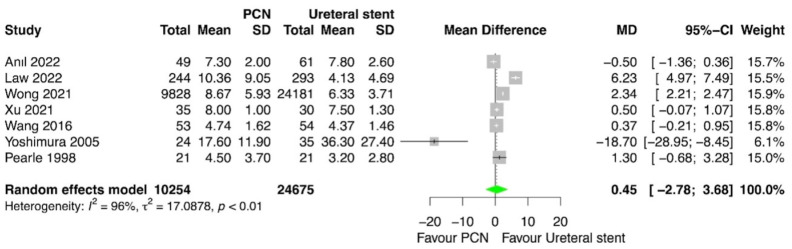
Forest plot: hospital length of stay [13,14,15,16,17,18,20].

The hospital length of stay was compared between PCN and retrograde ureteral stent placement in four studies [13,14,16,17]. The meta-analysis revealed no statistically significant difference between patients treated with PCN and those treated with retrograde ureteral stent placement for this outcome (*p* = 0.78; MD = 0.45; 95% CI = –2.78, 3.68; I^2^= 96%) (Figure 7).

### 3.8. Procedure Success Rate

**Figure 8:**
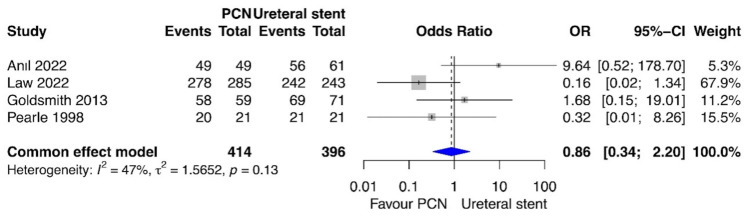
Forest plot: procedure success rate [17,18,19,20].

The procedure success rate was compared between PCN and retrograde ureteral stent placement in four studies [17,18,19,20]. The meta-analysis revealed no statistically significant difference between patients treated with PCN and those treated with retrograde ureteral stent placement for this outcome (*p* = 0.76; OR = 0.86; 95% CI = 0.34, 2.20; I^2^ = 47%) (Figure 8).

## 4. Discussion

Urolithiasis is a highly prevalent disease that is commonly encountered in the field of urology. This disorder has been examined in a limited number of epidemiologic studies in Asian countries. In a study conducted in Japan, Yasui et al. [21] found an annual rate of initial upper urinary tract stones of 134.0 per 100,000 individuals, which is lower than the reported incidence in Western nations. In South Korea, the annual incidence of urolithiasis is increasing [22]. Using the Korean National Health Insurance Service dataset, Tae et al. [23] calculated the cumulative incidence and lifetime prevalence of urolithiasis in the general population and evaluated the risk factors for urolithiasis. They found a yearly increase in annual incidence of urolithiasis (Poisson regression: hazard ratio, 1.025; *p* < 0.001) and an 11-year cumulative incidence of 5.71%. Among individuals diagnosed with urolithiasis, 21.3% developed recurrence within 5 years. Furthermore, the 11-year cumulative incidence of urolithiasis was higher in men than in women, with rates of 7.07% and 4.34%, respectively. The cumulative incidence over 11 years was highest among individuals in the 60–69 years age group (9.08%). The estimated standardized lifetime prevalence was 11.5% overall, with a higher rate in men than in women (12.9% versus 9.8%). The authors suggested that the increasing annual incidence of urolithiasis may be attributable to the increasing prevalence of comorbidities and alterations in lifestyle.

The field of PCN has advanced considerably since the era of William Goodwin, who unintentionally punctured the renal pelvis while performing translumbar aortography [24]. PCN has been traditionally performed by radiologists using fluoroscopic guidance, with success rates typically exceeding 95% [25]. Over the past decade, there has been a notable increase in the utilization of alternative guidance modalities, such as ultrasonography and computed tomography, for various medical procedures [26,27]. The use of sonographic guidance alone for PCN was first reported by Pedersen in 1974, with a reported success rate of 70% [28]. Since then, numerous studies have been conducted employing exclusively ultrasound guidance, with success rates as high as 92% [29]. The improvement in success rates may be attributed to the introduction of advanced ultrasound machines with high-resolution capabilities, enabling a clearer visualization of the pelvicalyceal system. Consequently, the success rate of procedures performed with ultrasound guidance has become comparable to that of procedures conducted under fluoroscopic guidance, while significantly eliminating the risks associated with radiation.

The first documented use of ureteral catheters was more than 100 years ago when Shoemaker provided the inaugural account of their use in women [30]. Since then, ureteral stents have been employed for a diverse range of urologic disorders. The utilization of an indwelling open-ended silicone ureteral stent for malignant obstruction was first described by Zimskind et al. [31] in 1967; however, this design tended to migrate. Gibbons et al. [32] developed a novel stent featuring a distal flange to mitigate the problem of proximal migration. In 1974, commercially available sharply pointed barbs were introduced to prevent distal migration [32]. In close succession, Finney and Hepperlen et al. [33,34] independently described a novel stent configuration for mitigating both proximal and distal migration. This design featured a J-shaped curl on each end of the stent and is now commonly referred to as the double-pigtail or double-J stent. The majority of recent stent designs are modifications of this model.

Obstructive uropathy is a prevalent disorder characterized by the presence of an anatomic or functional abnormality hindering the normal flow of urine [6]. Obstruction may occur at any location within the urinary tract. Obstructive uropathy is categorized according to the level of obstruction and the underlying cause, distinguishing between intrinsic and extrinsic factors affecting the urinary tract. The primary objective in managing acute urinary tract obstruction is to re-establish adequate urine drainage, either through the area of obstruction or by bypassing the obstruction. The need for immediate intervention depends on several factors, including the presence of a fever, the possibility of unresolved infection, uncontrollable pain from a renal colic, bilateral obstruction or the obstruction of a solitary kidney, the presence of high-grade obstruction, or the development of acute renal insufficiency. Individuals with obstructive pyelonephritis are particularly susceptible to developing urosepsis, along with its associated consequences, such as acute kidney injury and death [35,36]. The coexistence of an upper UTI and ureteral stones represents a critical situation necessitating urgent intervention. Prompt decompression, the administration of broad-spectrum antibiotics, and vigilant monitoring for the development of urosepsis are imperative. The reported mortality rates for patients diagnosed with obstructive pyelonephritis and sepsis are 9% for individuals who undergo decompression and 19% for those who do not undergo decompression [4]. Decompression through retrograde ureteral stent placement or PCN is essential to reduce both morbidity and mortality rates. PCN and retrograde ureteral stenting are both effective methods of decompression, although one method is preferred over the other in certain situations. For example, retrograde ureteral stenting is less effective when retrograde access is challenging or impossible, such as in patients with urinary diversion or previous renal transplantation. Retrograde ureteral stenting is also generally avoided when bladder access is difficult because of urethral stricture or lower-extremity contractures. PCN is contraindicated in patients with uncorrected coagulopathy or platelet dysfunction.

The EAU guidelines on urolithiasis recommend urgent decompression for sepsis or anuria in patients with upper urinary tract obstruction. Decompression is crucial to prevent additional complications in patients with infected hydronephrosis resulting from unilateral or bilateral urolithiasis-induced obstruction [8,9]. The guidelines also highlight the paucity of available evidence supporting the superiority of PCN over retrograde ureteral stent placement for the primary treatment of infected hydronephrosis and recommend that definitive stone removal be delayed until the infection is cleared following a complete course of antimicrobial therapy.

Several studies have compared PCN and retrograde ureteral stenting for obstructive uropathy. Zul Khairul Azwadi et al. [37] recently conducted a systematic review and meta-analysis comparing these two procedures. They included seven studies (encompassing a total of 667 patients) and found no discernible differences between PCN and retrograde ureteral stenting in terms of septic parameters, quality of life, failure rates, post-procedural pain, or utilization of analgesics. However, PCN treatment was associated with reduced rates of hematuria and dysuria following the procedure and a longer duration of hospitalization, compared to retrograde ureteral stents. Thus, PCN and retrograde ureteral stent placement were both efficacious for relieving urinary tract obstruction, but PCN may be preferred because of its minimal adverse effects on quality-of-life measures, specifically post-procedure hematuria and dysuria. The longer duration of hospitalization after PCN should be considered, although the difference in hospital stay between procedures is relatively small (MD, 1.82 days). Ramsey et al. [10] reviewed the literature regarding evidence for the optimal decompression method in patients with acute sepsis secondary to infected hydronephrosis. The authors found two RCTs comparing retrograde ureteral stent with PCN, one of which reported specifically on patients with acute sepsis and obstruction. Neither trial demonstrated differences between methods in terms of decompression or resolution of sepsis. When further reviewing the literature for complications associated with PCN versus retrograde ureteral stent placement, Ramsey et al. [10] found a general complication rate of 4% with PCN and noted that complications associated with retrograde ureteral stent procedures were inconsistently documented. They concluded that there was no substantial evidence supporting suggestions that retrograde stent placement may promote bacteremia or increase the overall risks in patients with acute urinary tract obstruction.

Several limitations of our study deserve consideration. First, the studies included in the meta-analyses did not uniformly meet the criteria for high-quality RCTs, resulting in a dearth of substantial evidence. Second, only three RCTs were included, with the remaining studies being retrospective and of high heterogeneity. Third, we were unable to analyze the complications associated with PCN or retrograde ureteral stenting. Although complications are important for clinical decision making, the included studies did not provide sufficient complication data for meta-analysis. Fourth, we did not consider the cost of the procedures. Cost-effectiveness analyses can be an important guide for both clinicians and patients. We anticipate that future investigations will be conducted to help deal with these limitations.

## 5. Conclusions

The clinical outcomes related to efficacy did not differ between PCN and retrograde ureteral stent placement when used for decompression in patients with severe urinary tract infection secondary to obstructive urolithiasis. Thus, the choice between procedures depends mainly on the urologist’s or patient’s preferences. However, insufficient evidence prevented analyses of adverse effects and costs, and future RCTs with large sample sizes are required to more conclusively determine the most appropriate method of decompression in this patient population.

## Figures and Tables

**Table 1:** Characteristics of the included studies.

Author Year	Country	Design	Procedure	Inclusion Criteria	Exclusion Criteria	No. Patients	Age, Years	Quality Assessment
Anıl et al., 2022 [20]	Turkey	Retrospective	PCN	Obstruction in the urinary system Fever (≥38 °C) Pyuria Costovertebral angle tenderness	Age < 18 y Solitary kidney Pyonephrosis Staghorn stones or multiple stones within the kidney Pregnancy Anticoagulant use Immune deficiency Sepsis Cancer	49	52.7 ± 13.1	2+
			Retrograde ureteral stent			56	48.0 ± 17.7	
Law et al., 2022 [18]	Singapore	Retrospective	PCN	Age ≥ 21 y CT confirmation of obstructive urolithiasis Temperature ≥ 38 °C and/or total WBC count ≥ 12,000 cells/mm^3^ within 24 h of surgical decompression	Urethral or ureteral stricture Urinary diversion Pregnancy Solitary kidney Noninfectious etiology of fever Elevated total WBC count not otherwise attributable to infection	244	58.02 ± 14.87	2+
			Retrograde ureteral stent			293	56.19 ± 15.22	
Wong et al., 2021 [15]	Canada	Retrospective	PCN	Age ≥ 18 y Diagnosis of sepsis and ureteral/renal calculi	Not stated	9828	64.8 ± 16.1	2−
			Retrograde ureteral stent			24,181	64.2 ± 16.0	
Xu et al., 2021 [14]	China	RCT	PCN	Upper urinary tract stones and urosepsis at admission Urosepsis was defined as an increase in the Sequential (sepsis-related) Organ Failure Assessment score by ≥2 points in the sepsis-3.0 evaluation	Urethral or ureteral stricture Urinary diversion Pregnancy Solitary kidney Severe sepsis (diagnosed as bacterial septic shock) Septic shock Unwillingness or inability to commit to the study’s follow-up protocol	35	65 (49–72)	1+
			Retrograde ureteral stent			30	64.5 (54–70)	
Wang et al., 2016 [16]	Taiwan	RCT	PCN	Obstructing ureteral stones and clinical signs of sepsis WBC count ≥ 10,000 cells/mm^3^ and/or temperature ≥ 38 °C	Urethral or ureteral stricture Urinary diversion Pregnancy Solitary kidney Severe sepsis or septic shock Unwillingness or inability to commit to the study’s follow-up protocol	53	58.2 ± 10.9	1+
			Retrograde ureteral stent			53	57.5 ± 11.9	
Goldsmith et al., 2013 [19]	USA	Retrospective	PCN	SIRS at the time of diagnosis (defined as ≥2 of the following: temperature > 38 °C or <36 °C, heart rate > 90 beats/min, respiratory rate > 20 breaths/min, WBC count > 12,000 cells/mm^3^ or <4000 cells/mm^3^, or ≥10% immature band forms) CT diagnosis of obstructive urolithiasis	Noninfectious indication for intervention (e.g., acute renal failure or uncontrolled pain in the absence of SIRS)	59	56 (19–88)	2+
			Retrograde ureteral stent			71		
Yoshimura et al., 2005 [13]	Japan	Retrospective	PCN	Upper urinary calculi SIRS Positive urine culture (>10^5^ cfu/mL) and no antibiotic therapy just prior to consultation Positive urine culture (>10^2^ cfu/mL) or pyuria (≥10 WBC cells/hpf in the centrifuged specimen with flank pain) and some antibiotic therapy just prior to consultation No clinical focus of infection other than the urinary tract	Acute prostatitis Acute epididymitis	24	59.5 ± 17.4	2−
			Retrograde ureteral stent			35	67.3 ± 15.7	
Pearle et al., 1998 [17]	USA	RCT	PCN	Obstructing ureteral or ureteropelvic junction stones Clinical signs of infection WBC count ≥ 17,000 cells/mm^3^ and/or temperature ≥ 38 °C	Uncorrected coagulopathy Urethral or ureteral stricture Urinary diversion Pregnancy Ureteral calculus > 15 mm Steinstrasse	21	41.3 ± 13.0	1+
			Retrograde ureteral stent			21	41.3 ± 14.5	

PCN, percutaneous nephrostomy; RCT, randomized controlled trial. The quality assessment was indicated by the Scottish Intercollegiate Guidelines Network checklist. 1+ means well conducted RCT with a low risk of bias. 2+ means well conducted cohort studies with a low risk of bias. 2− indicates cohort studies with a high risk of bias.

**Table 2:** MINORS scores of the nonrandomized studies.

	Anıl et al., 2022 [20]	Law et al., 2022 [18]	Wong et al., 2021 [15]	Goldsmith et al., 2013 [19]	Yoshimura et al., 2005 [13]
Clearly stated aim	2	2	2	2	2
Inclusion of consecutive samples	2	2	2	2	2
Prospective collection of data	0	0	0	0	0
Endpoints appropriate to the aim of the study	2	2	2	2	2
Unbiased assessment of the study endpoint	0	0	0	0	0
Follow-up period appropriate to the aim of the study	2	2	2	2	2
Loss to follow-up <5%	2	2	2	2	2
Prospective calculation of the study size	0	0	0	0	0
Adequate control group	2	2	2	2	2
Contemporary groups	2	2	2	2	2
Baseline equivalence of groups	2	2	2	2	1
Adequate statistical analyses	2	2	2	2	2
Total	18	18	18	18	17

Each item has received a score of 0 (not reported), 1 (reported but inadequate), or 2 (reported and adequate). The ideal (maximum) total MINORS score is 16 for noncomparative studies and 24 for comparative studies. MINORS, methodological index for nonrandomized studies.

## Data Availability

The data presented in this study are available in the article.

## Supplementary Materials

The following supporting information can be downloaded at https://www.mdpi.com/article/10.3390/medicina60060861/s1: Table S1: PRISMA Checklist; and Table S2: Search strategies.
