# Supplementary material for: Percutaneous Nephrostomy versus Ureteral Stent for Severe Urinary Tract Infection with Obstructive Urolithiasis: A Systematic Review and Meta-Analysis
Source: Medicina (Kaunas). 2024 May 24;60(6):861. doi: 10.3390/medicina60060861 (PMC11206041; doi:10.3390/medicina60060861)
Supplement: Supplementary file 1 [file medicina-60-00861-s001.zip › TableS2.docx]

**Table S2. Search Strategy**

| **PubMed** | | |
| --- | --- | --- |
| Search | Query | Items found |
| 1 | Percutaneous nephrostomy | 6,709 |
| 2 | PCN | 3,240 |
| 3 | #1 OR #2 | 9,656 |
| 4 | Ureteral stent | 7,397 |
| 5 | stenting | 134,010 |
| 6 | #4 OR #5 | 134,010 |
| 7 | Obstructive uropathy | 47,989 |
| 8 | urolithiasis | 44,396 |
| 9 | Ureter stone | 8,999 |
| 10 | #8 OR #9 | 45,232 |
| 11 | #3 AND #6 AND #7 AND #10 | 145 |
| ("nephrostomy, percutaneous"[MeSH Terms] OR ("nephrostomy"[All Fields] AND "percutaneous"[All Fields]) OR "percutaneous nephrostomy"[All Fields] OR ("percutaneous"[All Fields] AND "nephrostomy"[All Fields]) OR "PCN"[All Fields]) AND ((("ureter"[MeSH Terms] OR "ureter"[All Fields] OR "ureteral"[All Fields] OR "ureteric"[All Fields] OR "ureteritis"[All Fields]) AND ("stent s"[All Fields] OR "stentings"[All Fields] OR "stents"[MeSH Terms] OR "stents"[All Fields] OR "stent"[All Fields] OR "stented"[All Fields] OR "stenting"[All Fields])) OR ("stent s"[All Fields] OR "stentings"[All Fields] OR "stents"[MeSH Terms] OR "stents"[All Fields] OR "stent"[All Fields] OR "stented"[All Fields] OR "stenting"[All Fields])) AND (("obstruct"[All Fields] OR "obstructed"[All Fields] OR "obstructing"[All Fields] OR "obstruction"[All Fields] OR "obstructions"[All Fields] OR "obstructive"[All Fields] OR "obstructs"[All Fields]) AND ("urologic diseases"[MeSH Terms] OR ("urologic"[All Fields] AND "diseases"[All Fields]) OR "urologic diseases"[All Fields] OR "uropathy"[All Fields] OR "uropathies"[All Fields])) AND ("urolithiasis"[MeSH Terms] OR "urolithiasis"[All Fields] OR ("ureterolithiasis"[MeSH Terms] OR "ureterolithiasis"[All Fields] OR ("ureter"[All Fields] AND "stone"[All Fields]) OR "ureter stone"[All Fields])) | | |
| **EMBASE** | | |
| Search | Query | Items found |
| 1 | Percutaneous nephrostomy | 8,626 |
| 2 | Ureter stent | 14,824 |
| 3 | Ureteral stenting | 3,248 |
| 4 | #2 OR #3 | 15,811 |
| 5 | Obstructive uropathy | 5,391 |
| 6 | Ureter stone | 18,132 |
| 7 | #4 AND #5 AND #6 | 82 |
| **Cochrane library** | | |
| Search | Query | Items found |
| 1 | Percutaneous nephrostomy | 521 |
| 2 | PCN | 216 |
| 3 | #1 OR #2 | 707 |
| 4 | Ureteral stent | 760 |
| 5 | stenting | 5,749 |
| 6 | #4 OR #5 | 6,302 |
| 7 | Obstructive uropathy | 80 |
| 8 | urolithiasis | 915 |
| 9 | Ureter stone | 1,225 |
| 10 | #8 OR #9 | 1,945 |
| 11 | #3 AND #6 AND #7 AND #10 | 1 |
| **Web of Science** | | |
| Search | Query | Items found |
| 1 | Percutaneous nephrostomy | 2,572 |
| 2 | PCN | 5,617 |
| 3 | #1 OR #2 | 7,998 |
| 4 | Ureteral stent | 1,429 |
| 5 | stenting | 36,539 |
| 6 | #4 OR #5 | 37,670 |
| 7 | Obstructive uropathy | 1,940 |
| 8 | urolithiasis | 10,891 |
| 9 | Ureter stone | 2,378 |
| 10 | #8 OR #9 | 12,716 |
| 11 | #3 AND #6 AND #7 AND #10 | 1 |
| **Google Scholar** | | |
| Search | Query | Items found |
| 1 | ("Percutaneous nephrostomy" OR "PCN") AND ("Ureteral stent" OR "Stenting") AND "Obstructive uropathy" AND ("urolithiasis" OR "ureter stone") | 592 |
